# The Roles of EETs in Neurological Disorders

**DOI:** 10.1002/cns.71121

**Published:** 2026-09-08

**Authors:** Rong Tang, Mengya Li, Banglian Hu, Guoqiang Fei, Honghua Zheng, Xin Wang, Jing Ding

**Affiliations:** ^1^ Department of Neurology Zhongshan Hospital (Xiamen), Fudan University Xiamen Fujian China; ^2^ Fujian Provincial Key Laboratory of Neurodegenerative Disease and Aging Research, Institute of Neuroscience School of Medicine, Xiamen University Xiamen Fujian China; ^3^ Department of Neurology Zhongshan Hospital, Fudan University Shanghai China

**Keywords:** EETs, neuroinflammation, neurological disorders, sEH, sEH inhibitors

## Abstract

**Background:**

Epoxyeicosatrienoic acids (EETs) are bioactive lipid mediators derived from arachidonic acid (ARA) via cytochrome P450 (CYP450) enzymes. They exert pleiotropic effects including anti‐inflammation, anti‐apoptosis, antioxidant activity, and vasodilation. Soluble epoxide hydrolase (sEH) rapidly hydrolyzes EETs to inactive diols, and its inhibition has emerged as a promising strategy to potentiate EET‐mediated benefits. This review synthesizes current evidence on the roles and mechanisms of EETs and sEH in major neurological disorders and discusses translational challenges and future directions.

**Methods:**

A narrative review was conducted to synthesize evidence on the biosynthesis and metabolism of EETs, the expression and regulation of brain cytochrome P450 epoxygenases and sEH, the neuroprotective effects of EETs, and the therapeutic potential of sEH inhibitors, and to identify current limitations and future strategies for sEH‐targeted therapies.

**Results:**

EETs ameliorate multiple neurological disorders through anti‐inflammatory and anti‐apoptotic effects, increased cerebral blood flow, reduced excitotoxicity, decreased dendritic spine loss, reduced oxidative stress, and enhanced neurosteroid secretion. In animal models, sEH inhibition elevates endogenous EET bioavailability, slows progression of various central nervous system diseases, and attenuates brain injury. However, sEH inhibitor development faces several challenges, including species differences in CYP expression, insufficient blood–brain barrier penetration, bleeding risks, failure of some candidates in late‐stage trials, and the limitations of single‐target inhibition.

**Conclusions:**

EETs are endogenous multifaceted neuroprotective mediators that act through convergent anti‐inflammatory, anti‐apoptotic, and vascular mechanisms. sEH inhibition represents a highly attractive yet challenging therapeutic strategy for a wide spectrum of central nervous system disorders. Future progress requires: development of brain‐penetrant or dual‐target sEH inhibitors; identification of EET‐specific G protein‐coupled receptors (GPCRs) and biomarkers for patient stratification; improved preclinical models that better translate to humans; and rigorous clinical evaluation to define safety and efficacy.

## Graphical abstract

Epoxyeicosatrienoic acids (EETs), derived from arachidonic acid (ARA) via cytochrome P450 (CYP450) enzymes, are produced in nearly all human tissues and exert a wide range of beneficial effects. This review summarizes the roles and potential mechanisms of EETs in neurological disorders, with a focus on cerebrovascular diseases, epilepsy, Parkinson's disease, Alzheimer's disease, psychiatric conditions, neuropathic pain, and preliminarily discusses some noteworthy issues and future research directions faced by EETs in drug development.

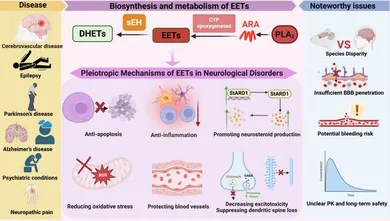

## 1 Introduction

Epoxyeicosatrienoic acids (EETs), metabolites of arachidonic acid (ARA) generated by cytochrome P450 (CYP450) enzymes, are widely distributed across various human tissues—notably in blood vessels, brain tissue, and the cardiovascular system. EETs exert a range of beneficial effects through multiple signaling pathways, including anti‐inflammatory, anti‐apoptotic, and antioxidant actions, as well as alleviation of endoplasmic reticulum stress. These functions underscore their broad therapeutic potential, particularly in cardiovascular diseases and cancer. In recent years, growing research has highlighted the significance of EETs in neurological disorders, emphasizing their role in modulating neuronal activity, attenuating neuroinflammation, regulating cerebral blood flow, and enhancing the survival of neuronal and glial cells. This review summarizes the roles and potential mechanisms of EETs in a spectrum of neurological conditions, including cerebrovascular disease, epilepsy, Parkinson's disease, Alzheimer's disease, psychiatric disorders, neuropathic pain, and preliminarily discusses some noteworthy issues and future research directions faced by EETs in drug development.

## 2 Biosynthesis and Metabolism of EETs

Arachidonic acid (ARA), a polyunsaturated fatty acid, is cleaved from membrane phospholipids by phospholipase A2 (PLA2) in response to physiological stimuli or pathological injury. Through subsequent cascade reactions, ARA gives rise to a variety of endogenous signaling molecules that regulate numerous biological processes. The metabolism of ARA primarily proceeds through three enzymatic pathways: the cyclooxygenase (COX) pathway, the lipoxygenase (LOX) pathway, and the cytochrome P450 (CYP450) pathway [1].

The COX pathway generates prostaglandins (PGs), which are involved in inflammation, platelet aggregation, cardiovascular disease, pain, and cancer. LOX metabolites, including leukotrienes, lipoxins, and hydroxyeicosatetraenoic acids (HETEs), have been extensively studied in the context of asthma, pulmonary diseases, and atherosclerosis [2, 3, 4]. In contrast, the CYP450 pathway was not identified until the early 1980s and encompasses both hydroxylase and epoxygenase activities [5]. CYP hydroxylases produce HETEs such as 20‐HETE, which exhibit proinflammatory and vasoconstrictive properties, whereas CYP epoxygenases generate epoxyeicosatrienoic acids (EETs), which mediate a spectrum of beneficial effects [6, 7].

**FIGURE 1:**
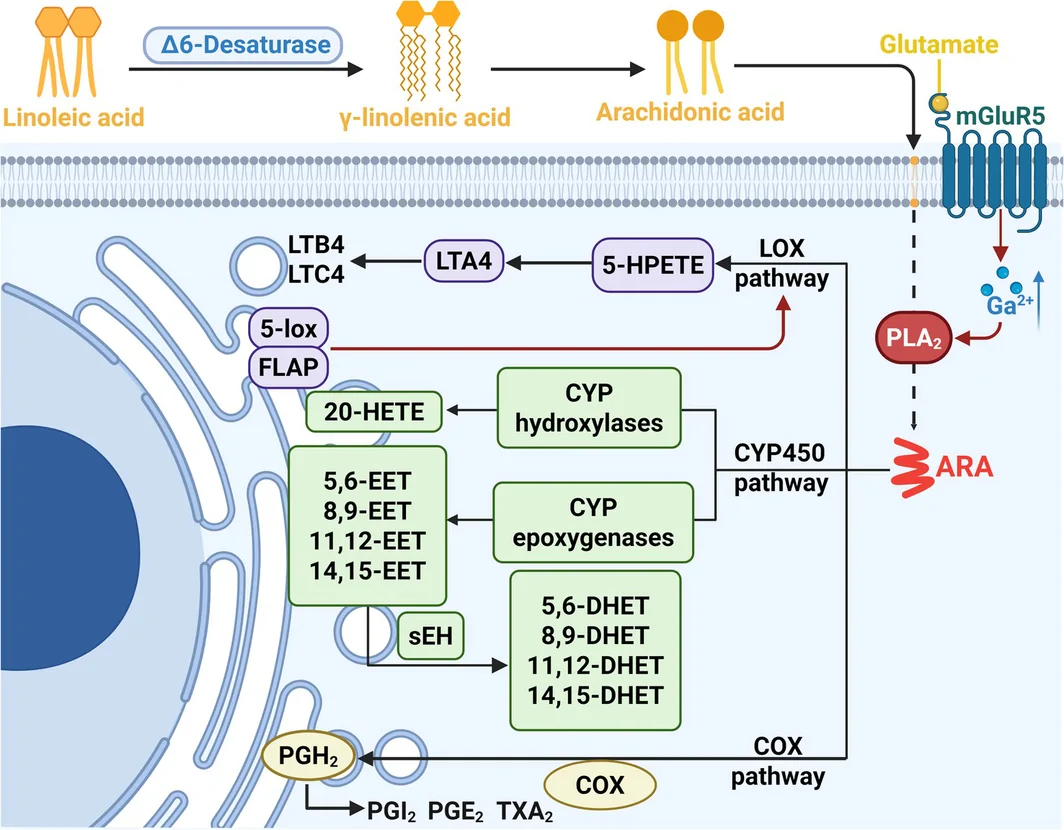
Biosynthesis and metabolism of epoxyeicosatrienoic acids (EETs) within the arachidonic acid (ARA) cascade. The arachidonic acid (ARA) cascade proceeds primarily through three metabolic pathways: Cyclooxygenase (COX), lipoxygenase (LOX), and cytochrome P450 (CYP450). The COX pathway yields prostaglandins (PGs), whereas the LOX pathway produces metabolites such as leukotrienes, lipoxins, and hydroxyeicosatetraenoic acids (HETEs). The CYP450 pathway, which includes both hydroxylase and epoxygenase activities, generates HETEs—for example, 20‐HETE, which exhibits pro‐inflammatory and vasoconstrictive properties—as well as a series of beneficial epoxyeicosatrienoic acids (EETs). EETs comprise four regioisomers (14,15‐EET, 11,12‐EET, 8,9‐EET, and 5,6‐EET), which are metabolized by soluble epoxide hydrolase (sEH) into dihydroxyeicosatrienoic acids (DHETs), leading to the loss of their biological activity.

The four EET regioisomers (14,15‐EET, 11,12‐EET, 8,9‐EET and 5,6‐EET) elicit distinct physiological responses across different tissues, including vasodilation, anti‐inflammation, anti‐apoptosis, antioxidant activity, as well as promotion of angiogenesis and fibrinolysis [1, 2, 8]. EETs are metabolized by soluble epoxide hydrolase (sEH) to dihydroxyeicosatrienoic acids (DHETs), a process that inactivates their biological activity [2]. The biosynthesis and metabolism of EETs within the ARA cascade are depicted in Figure 1. Both genetic deletion and pharmacological inhibition of sEH have been demonstrated to significantly preserve or elevate endogenous EET levels, yielding beneficial outcomes in experimental models of cardiovascular disease, diabetes, inflammatory disorders, pain, pulmonary disease and cancer [9, 10].

## 3 Metabolism and Distribution of EETs and sEH in the Brain

Previous studies have confirmed the presence of endogenous EETs in the brain, with their synthesis observed in specific cell types—including astrocytes, cerebral vascular endothelial cells, and neurons—in rodent models [5, 11, 12]. Furthermore, cytochrome P450 epoxygenases (CYPs), the enzymes that produce EETs, are expressed in multiple brain regions and are localized to both vascular and non‐vascular compartments [5, 13]. Consistent with this distribution, CYP epoxygenases are found throughout the central nervous system (CNS) in neurons, astrocytes, and vascular endothelial cells, supporting the view that EETs mediate cellular signaling and contribute to diverse biological processes in the CNS [14].

The principal metabolic pathway for EETs in the brain is hydrolysis by sEH to their corresponding diols, which can exert pro‐inflammatory effects [1]. When sEH activity is suppressed—via downregulation or inhibition—EETs may be partially metabolized through β‐oxidation into 16‐carbon species or chain‐elongated to form bioactive 22‐carbon metabolites [15]. sEH is widely expressed in human tissues, most prominently in the liver, kidney, intestine, and vasculature, and is also highly expressed in the brains of both rodents and humans. At the cellular level, sEH localizes primarily in the cytoplasm of neurons, astrocytes, oligodendrocytes, and the smooth muscle and endothelial cells of cerebral small arteries, as well as in nerve fibers innervating cortical surface arteries [16, 17, 18, 19].

The co‐localization of EET‐synthesizing enzymes and sEH across diverse brain cell types suggests a coordinated system wherein some cells primarily produce EETs while others are specialized in their response and metabolism. This arrangement implies that sEH critically regulates local levels of bioactive EETs, thereby influencing cerebral circulation, neural activity, and the pathophysiology of neurological disorders [10, 16].

Notably, sEH expression exhibits sexual dimorphism. Hepatic and renal sEH levels are higher in male mice than in females [20], a difference attributed to hormonal regulation, particularly by estrogens [21]. This sex‐dependent expression extends to the brain, where higher sEH levels in males correspond to elevated plasma 14,15‐DHET levels [22]. The sex‐differential expression of sEH in cortical neurons and endothelial cells further suggests its potential impact on cerebrovascular function [23].

## 4 Brain Cytochrome P450 Epoxygenases: Expression, Function, and Drug Discovery Implications

Cytochrome P450 (CYP) epoxygenases in the brain are key enzyme systems in the arachidonic acid metabolic pathway responsible for generating EETs. Although total CYP content in the brain is only approximately 0.5%–2% of that in the liver [24], the unique expression patterns and functional significance of brain CYPs make them compelling targets for drug discovery.

### 4.1 Human Brain CYP Epoxygenases

Multiple CYP epoxygenases are expressed in the human brain. Among these, the CYP2J and CYP2C subfamilies are the primary contributors directly involved in EETs biosynthesis. CYP2J2 is the predominant CYP epoxygenase expressed in the human brain [25]. It is widely distributed across multiple brain regions, including the cerebral cortex, hippocampus, basal ganglia, amygdala, and cerebellum. In situ hybridization studies have shown that *CYP2J2* mRNA is localized in specific brain regions such as the cortex, lateral ventricular area, and olfactory cortex [26]. The CYP2C subfamily also holds an important position in the human brain. CYP2C8, CYP2C9, and CYP2C19 are all expressed in the brain with distinct expression patterns and functions. CYP2C9 and CYP2C19 have been detected in microsomal fractions from multiple human brain regions, including the frontal cortex, hippocampus, basal ganglia, amygdala, and cerebellum, and are primarily localized to neuronal soma, with expression extending to axons and dendrites in some regions [27]. CYP2C19 is particularly notable for its expression in the developing fetal brain, where it is approximately sixfold higher than in fetal liver, suggesting important roles in neurodevelopment [28]. Although present at lower levels than CYP2C9 and CYP2C19 in the brain, *CYP2C8* mRNA has been detected in multiple brain regions, with expression observed in the supraoptic nucleus and along the ventral brain surface, and it remains a contributor to brain arachidonic acid epoxidation [26].

### 4.2 Rodent Brain CYP Epoxygenases

The expression profiles of CYP epoxygenases in rodent (mouse and rat) brains differ substantially from those in humans. Understanding these differences is crucial for translational research. The Cyp2j subfamily is highly diverse in the mouse brain. Studies show that the mouse Cyp2j subfamily comprises seven members (Cyp2j5, Cyp2j6, Cyp2j8, Cyp2j9, Cyp2j11, Cyp2j12, Cyp2j13). Among these, Cyp2j9 and Cyp2j12 are highly expressed in mouse brain, with Cyp2j12 showing particularly brain‐enriched expression [29]. The Cyp2c subfamily in mice includes up to 15 members and is a major contributor to brain EETs production [30]. Studies indicate that LPS‐induced inflammation downregulates the expression of epoxygenases such as Cyp2c11 and Cyp2j3 in the rat brain, suggesting that inflammatory states exert significant regulatory control over brain CYP epoxygenase activity [31]. Rat brain CYP expression exhibits pronounced sexual dimorphism. Gerges and EI‐Kadi conducted a systematic comparison of P450 enzyme expression in the heart, liver, kidney, lung, brain, and small intestine of both male and female Sprague–Dawley rats, identifying multiple CYP isoforms with sex‐specific expression patterns. In the liver, Cyp2c11, Cyp2c13, and Cyp4a2 showed male‐specific high expression, whereas Cyp2c12 showed female‐specific expression. In the brain, enzymes such as CYP1A2, CYP3A, and CYP4A1 displayed a trend toward higher expression in females across multiple organs, including the liver [32]. These findings underscore that sex effects must be carefully considered when translating rodent study findings to humans.

### 4.3 Relevance to Drug Discovery

Brain CYP epoxygenases represent an underexplored frontier in CNS drug discovery. While sEH inhibition is currently the more clinically advanced approach, direct targeting of CYP epoxygenases—combined with a deep understanding of species‐specific expression patterns and genetic variability—offers promising avenues for developing novel therapeutics for neurological and psychiatric disorders. The marked species differences between humans and rodents underscore the necessity of using humanized models and region‐specific analyses to accelerate successful clinical translation (Table 1).

**TABLE 1:** Key brain CYP epoxygenases and drug discovery implications.

Species	CYP isoforms	Key features	Drug discovery implications
Human	CYP2J2	Major brain epoxygenase; among top six most abundant brain P450s [33]	Target for EET enhancement; drug metabolism
	CYP2C8,2C9, 2C19	Fatty acid epoxygenation; genetic polymorphisms [27, 28]	Variable drug response; neurosteroid metabolism
	CYP2U1	Among top six most abundant brain P450s [33]	EET production; function under investigation
	CYP1B1	Among top six most abundant brain P450s [33]	EET production; xenobiotic metabolism
	CYP2E1	Among top six most abundant brain P450s; novel neuroinflammation target [33, 34]	Direct drug target for neuroinflammation
	CYP2D6	Polymorphic; among top six most abundant brain P450s [33]	Individualized CNS therapy
	CYP46A1	Most abundant brain P450 [33]; cholesterol 24‐hydroxylase [35]	Alzheimer's disease target (validated)
	CYP4X1	Highly brain‐enriched orphan P450 [33]	Function under investigation
Mouse	Cyp2j (7 isoforms: 2j5,6,8,9,11,12,13)	Mouse‐specific expansion; Cyp2j9 and Cyp2j12 brain‐high [29]	Model for human CYP2J2—caution needed
	Cyp2c (15 members)	Expanded family; inflammatory regulation [30]	Species differences limit translation
Rat	Cyp4x1	Highly brain‐enriched [36]	Orphan target
	Cyp2c11	Male‐specific expression [32]	Sexual dimorphism caution
	Cyp2j10	Brain‐biased expression [37]	Brain‐enriched target potential
	Cyp2j3,2j4	Major epoxygenases; downregulated by inflammation [31]	Inflammation studies

Key future research priorities include: (1) developing brain‐region‐specific or cell‐type‐specific CYP modulators based on recent human brain mapping data; (2) exploring the newly identified roles of CYP2E1 in neuroinflammation as a direct drug target; (3) elucidating the functions of orphan brain‐enriched CYPs such as CYP4X1; (4) advancing humanized CYP mouse models to bridge the translational gap; and (5) validating CYP‐derived eicosanoids as clinical biomarkers for patient stratification in CNS drug trials.

## 5 sEH Inhibitors (sEHIs)

sEH inhibitors (sEHIs) are a class of pharmacological agents that inhibit soluble epoxide hydrolase (sEH) activity, thereby prolonging the biological effects of epoxyeicosatrienoic acids (EETs). These compounds exert anti‐inflammatory, antioxidant, and vasodilatory actions, and have demonstrated promising therapeutic potential in the treatment of cardiovascular, neurodegenerative, and metabolic diseases. Since their initial development, a diverse array of sEHI structural classes has emerged, each possessing distinct advantages and limitations, as summarized below for the principal compounds.

### 5.1 AUDA

AUDA (12‐(3‐adamantan‐1‐yl‐ureido)‐dodecanoic acid) is a potent sEHI that exhibits significant antihypertensive and anti‐inflammatory effects and has demonstrated cardiovascular protective effects in various animal models [38]. AUDA markedly reduces vascular permeability and the expression of inflammatory mediators and can protect against ischemia–reperfusion injury [39]. However, AUDA has poor water solubility, low oral bioavailability, and limited metabolic stability, which restrict its clinical utility [38].

### 5.2 AEPU

AEPU (N‐adamantyl‐N′‐(2‐(2‐ethoxyethoxy)ethyl)‐urea) shows good antihypertensive and anti‐inflammatory effects, and its oral bioavailability and water solubility are improved relative to AUDA [40]. But AEPU has lower selectivity and may inhibit off‐target enzymes, leading to potential side effects [41].

### 5.3 t‐AUCB

t‐AUCB (trans‐4‐[4‐(3‐adamantan‐1‐yl‐ureido)‐cyclohexyloxy]‐benzoic acid) is one of the earlier developed sEHIs and displays anti‐inflammatory, anticancer and neuroprotective properties [42]. It can effectively enhance the anti‐inflammatory effects of nonsteroidal anti‐inflammatory drugs (NSAIDs)—for example, showing improved anti‐inflammatory efficacy when combined with aspirin or other anti‐inflammatory agents [43]. However, t‐AUCB has relatively poor metabolic stability, a short half‐life and low bioavailability, necessitating frequent dosing to maintain efficacy [38]. In some tumor models, it may increase resistance by inhibiting the activity of apoptosis‐related proteins [44].

### 5.4 TPPU

TPPU (1‐(1‐propanoylpiperidin‐4‐yl)‐3‐[4‐(trifluoromethoxy)phenyl]urea) has high metabolic stability and can maintain in vivo activity for extended periods, thereby prolonging the biological effects of EETs [45]. It shows favorable anti‐inflammatory, antioxidant and antihypertensive effects and has demonstrated notable neuroprotection in animal models [46]. As a bifunctional agent, TPPU can also inhibit p38 kinase, further enhancing its anti‐inflammatory and neuroprotective effects [47]. It has relatively high bioavailability, and oral dosing can achieve systemic distribution and stable drug levels [48]. However, long‐term use of TPPU may lead to accumulation of metabolites, and its complex metabolic pathways require further study to clarify the potential physiological effects of its metabolites [49].

Each sEHI offers certain advantages in anti‐inflammatory, antihypertensive, and neuroprotective applications, but they differ in metabolic stability, bioavailability, and selectivity (Table 2). Selecting an appropriate sEHI according to specific clinical goals can optimize therapeutic outcomes and reduce the risk of adverse effects.

**TABLE 2:** The merits and drawbacks of the primary sEHI.

Classification	Principal researcher	Discovery year	Merits	Drawbacks
AUDA	Bruce D. Hammock and his team [50]	2005 [50]	Significant antihypertensive, anti‐inflammatory effects, demonstrated cardiovascular protective effects [38]; Protect against ischemia–reperfusion injury [39]	Poor water solubility, low oral bioavailability, limited metabolic stability [38]
AEPU	Bruce D. Hammock and his team [51]	2008 [51]	Good antihypertensive, good anti‐inflammatory effects, higher oral bioavailability and water solubility compared to AUDA [40]	Lower selectivity, may inhibit off‐target enzymes [41]
t‐AUCB	Bruce D. Hammock and his team [42, 52]	2007 [52]	Anticancer, neuroprotective properties, anti‐inflammation [42]; Effectively enhance the anti‐inflammatory effects of nonsteroidal anti‐inflammatory drugs [43]	Poor metabolic stability, a short half‐life, low bioavailability [38]; May increase resistance by inhibiting the activity of apoptosis‐related proteins [44]
TPPU	Bruce D. Hammock and his team [45, 53]	2013 [45, 53]	High metabolic stability [45]; Favorable anti‐inflammatory, favorable antioxidant, favorable antihypertensive effects, demonstrated notable neuroprotection in animal models [46]; Inhibit p38 kinase [47]; Relatively high bioavailability [54]	Accumulation of metabolites [54]

In recent years, significant progress has been made in research on sEH inhibitors in animal models of neurological diseases, and several novel candidates have advanced into clinical development. Long‐term TPPU treatment has been widely validated to improve cognitive function in multiple Alzheimer's disease (AD) models [55, 56, 57, 58]. UB‐SCG‐74 shows sustained efficacy (4 weeks post‐treatment) and superior performance to donepezil in 5XFAD mice. Safety pharmacology studies showed no toxicity, supporting advancement into preclinical regulatory evaluation [59]. While EC5026 has advanced into Phase 1b clinical trials for PD (STEP Study, NCT07142044) and neuropathic pain (NCT06438471), marking a critical stage in the translation of sEH inhibitors from the laboratory to clinical application.

Nevertheless, the translational path still faces many challenges: insufficient BBB penetration, potential bleeding risk, efficacy variability due to species differences, and uncertainties regarding pharmacokinetic properties and long‐term safety. Future research should focus on: developing next‐generation sEH inhibitors with high BBB penetration; identification and validation of clinical biomarkers (EET/DHET ratios, sEH activity) to guide patient stratification; thoroughly evaluating the coagulation risk of sEH inhibition and establishing appropriate risk management protocols; exploration of dual‐target inhibitors (sEH/FAAH, sEH/COX‐2) for synergistic effects; and strengthening cross‐species validation from rodent models to large animals and humans.

## 6 EETs in Cerebrovascular Disease

A growing body of evidence indicates that EETs mediate crucial protective effects in cerebrovascular diseases—such as ischemic stroke and intracerebral hemorrhage—through multiple mechanisms. These mechanisms include: (1) Anti‐apoptotic and antioxidant actions: 14,15‐EET significantly reduces ischemic brain injury by inhibiting cerebral microvascular smooth muscle cell apoptosis under oxygen–glucose deprivation via the JNK/c‐Jun and mTOR pathways, thereby lowering ROS production and enhancing cell survival [60, 61]. It also promotes astrocytic BDNF release, further supporting neuroprotection [60]. The other two studies further extended the neuroprotective mechanisms of 14,15‐EET by demonstrating the inhibition of neuronal parthanatos (a PARP‐1‐dependent programmed cell death) and pyroptosis [62, 63]. (2) Regulation of neurovascular function: EETs induce vasodilation and increase local cerebral blood flow, thereby improving nutrient supply. They also modulate calcium channels and vascular smooth muscle tone, which enhances cerebrovascular reactivity and permeability [64, 65]. (3) Anti‐inflammatory effects: By suppressing the release of pro‐inflammatory mediators and reducing oxidative stress, EETs help mitigate neuroinflammation after intracerebral hemorrhage and decrease neuronal cell death through the activation of PPAR‐α or the inhibition of the NF‐κB pathway [66, 67, 68, 69]. In summary, EETs provide multi‐faceted neuroprotection by suppressing inflammation and oxidative stress, limiting secondary injury, preventing apoptosis, and improving neurovascular function. Extending their half‐life to enhance bioactivity thus represents a promising therapeutic strategy for cerebrovascular disorders.

## 7 EETs in Cerebral Small Vessel Disease

Cerebral small vessel disease (CSVD) has garnered increasing attention due to its significant role in ischemic stroke and vascular cognitive impairment (VCI). Accounting for approximately 25% of ischemic strokes and representing the leading cause of VCI [70], CSVD is a major source of functional decline, disability, and cognitive deficits in the elderly. Diagnosis is primarily based on neuroimaging features, such as subcortical microinfarcts, lacunar infarcts, white matter hyperintensities (WMH), enlarged perivascular spaces, and microbleeds [71].

Growing evidence links soluble epoxide hydrolase (sEH) activity to VCI pathogenesis. In postmortem studies, sEH immunoreactivity is elevated in the vascular endothelium near microinfarcts and WMH lesions, accompanied by increased levels of its diol metabolite, 14,15‐dihydroxyeicosatrienoic acid (14,15‐DHET) [72]. Clinically, a higher serum ratio of polyunsaturated fatty acid diols to their epoxide precursors—reflecting sEH activity—has been associated with periventricular WMH in VCI patients [73]. These observations suggest that increased sEH activity and the subsequent reduction in bioactive epoxyeicosatrienoic acids (EETs) contribute to CSVD and related cognitive impairment.

Further studies indicate that EETs may protect against CSVD progression by promoting cerebral vasodilation and regulating blood flow. EETs play a key role in cerebral autoregulation through endothelial‐neural interactions [40]. Specifically, 14,15‐EET attenuates ischemia–reperfusion injury in brain microvascular endothelial cells via the SIRT1/FOXO3a pathway and translocator protein (TSPO), thereby mitigating vascular injury‐driven neuroinflammation and apoptosis [74]. Moreover, low levels of sEH‐metabolized linoleic acid products correlate with CSVD imaging markers, including WMH and microbleeds. Importantly, sEH inhibition has been shown to enhance EET bioactivity and may slow CSVD progression [75].

In summary, EETs exert multiple protective effects in CSVD, including regulation of cerebral blood flow, attenuation of ischemic injury, anti‐inflammatory and antioxidant actions, and modulation of metabolic pathways. Therefore, strategies aimed at prolonging EET bioactivity represent a promising therapeutic approach to decelerate the development of CSVD.

## 8 EETs in Epilepsy

Epilepsy is a common chronic neurological disorder characterized by recurrent seizures resulting from excessive, hypersynchronous firing of neurons in the brain. Growing evidence suggests that epoxyeicosatrienoic acids (EETs) play a role in alleviating seizure activity. Studies indicate that EETs, particularly 11,12‐EET, significantly reduce the excitability of hippocampal CA1 pyramidal neurons by activating G‐protein‐coupled inwardly rectifying potassium (GIRK) channels. This leads to decreased glutamate release and suppressed epileptiform activity—an effect that persists even in the presence of the functional antagonist EEZE, underscoring the therapeutic potential of EETs in epilepsy [76].

During seizures, large quantities of polyunsaturated fatty acids such as arachidonic acid are released. EETs can counteract this hyperexcitability not only via potassium channels but also through GABA‐related mechanisms. Inhibition of soluble epoxide hydrolase (sEH), which degrades EETs, prolongs their half‐life and has been shown to delay seizure onset—a process potentially linked to enhanced neurosteroid synthesis [77]. For instance, the sEH inhibitor TPPU potentiates the anticonvulsant effects of EETs. Furthermore, sEH inhibition and 14,15‐EET administration have been found to improve seizure‐induced cognitive deficits by enhancing hippocampal long‐term potentiation (LTP). This protection involves modulation of the cAMP and CaMKII signaling pathways, leading to improved function of NMDA and AMPA receptors [78].

EETs also mitigate seizure severity through anti‐inflammatory mechanisms. They attenuate neuroinflammation during epileptic episodes, particularly by reducing glutamate‐mediated excitotoxicity and associated neuronal death. This anti‐inflammatory property, which is consistently observed across neurodegenerative contexts, further supports the potential of EETs as antiepileptic agents [79]. In experimental models, EETs exhibit neuroprotective effects by reducing seizure‐induced dendritic spine loss and inflammatory responses. Pretreatment with TPPU not only decreases seizure severity but also ameliorates comorbid depression‐like behaviors, correlating with suppressed microglial activation and lower levels of inflammatory mediators such as IL‐1β [80].

In summary, EETs counter epilepsy through multiple synergistic mechanisms: reducing neuronal excitability, enhancing GABA‐mediated inhibition, improving synaptic plasticity, preventing dendritic spine loss, and suppressing neuroinflammation. These findings highlight sEH inhibition as a promising novel therapeutic strategy for epilepsy.

## 9 EETs in Parkinson's Disease

Parkinson's disease (PD) is a multifactorial neurodegenerative disorder driven by processes such as oxidative stress, neuroinflammation, and apoptosis. EETs counter these pathological mechanisms by modulating multiple signaling pathways, thereby reducing oxidative and endoplasmic reticulum stress, and subsequently suppressing neuroinflammatory and apoptotic responses. These actions help alleviate PD‐related neuronal damage. Consequently, inhibiting the EET‐metabolizing enzyme soluble epoxide hydrolase (sEH) to prolong EET half‐life has emerged as a potential therapeutic strategy for PD [81, 82].

Among EETs, 14,15‐EET—a major epoxy fatty acid—has shown pronounced neuroprotective effects in PD models. sEH expression is elevated in the brains of PD patients, and either pharmacological inhibition of its hydrolase domain or genetic deletion of sEH in mice attenuates the neurotoxicity induced by 1‐methyl‐4‐phenyl‐1,2,3,6‐tetrahydropyridine (MPTP). By preventing 14,15‐EET degradation, these approaches raise brain EET levels, curb neuroinflammation, and slow neurodegeneration, suggesting a promising direction for neuroprotective drug development in PD [83, 84].

EETs also protect neurons by countering oxidative stress‐induced apoptosis in PD. Given that dopaminergic neuron loss in PD is closely associated with oxidative injury, 14,15‐EET has been found to reduce PTEN oxidation and modulate Akt phosphorylation, thereby attenuating pro‐apoptotic signaling and promoting neuronal survival [85].

Therefore, EETs exert neuroprotection in PD primarily through anti‐oxidation, anti‐apoptosis, and anti‐inflammation [86, 87]. Stabilizing EETs via sEH inhibition has been validated in multiple PD models (MPTP‐treated mice, rotenone‐treated N27 cells, and Drosophila), with key molecular mediators including NF‐κB, Akt, and JNK/c‐Jun pathways [88, 89, 90]. These findings support sEH as a promising therapeutic target for PD.

## 10 EETs in Alzheimer's Disease or Cognitive Impairment

Alzheimer's disease (AD) is the most prevalent neurodegenerative disorder associated with dementia. Recent studies indicate that epoxyeicosatrienoic acids (EETs) contribute to neuroprotection, anti‐inflammation, and the amelioration of mitochondrial dysfunction in AD. Pretreatment with EETs has been shown to effectively alleviate Aβ‐induced mitochondrial impairment. In cellular AD models, EETs help maintain mitochondrial membrane potential, reduce mitochondrial fragmentation, and enhance cellular oxygen consumption. Exogenous EET supplementation significantly attenuates Aβ‐induced reactive oxygen species (ROS) production, mitigates mitochondrial dysfunction, lowers oxidative stress, and improves cellular respiration, thereby reducing Aβ neurotoxicity and counteracting disease progression [91]. Furthermore, TPPU effectively attenuated astrocytic and microglial activation, protected hippocampal neurons and synaptic proteins, and ultimately improved cognitive function [57], providing a rationale for developing EET‐based AD therapies.

Moreover, sEH inhibition alleviates neuroinflammation, neuronal death, and oxidative stress by stabilizing endogenous EET levels—particularly 8,9‐EET and 14,15‐EET. In Aβ‐induced AD mouse models, this leads to anti‐AD effects via modulation of GSK3β‐regulated NF‐κB, p53, and Nrf2 signaling pathways [92]. Consistent with this, in both a Drosophila AD model and human co‐culture cell models, TPPU significantly suppressed neuroinflammation by elevating 14,15‐EET levels and inhibiting the TLR4/NF‐κB and p38 MAPK pathways, and improved olfactory memory and learning abilities in flies [93]. These findings further validate the sEH pathway as a cross‐species therapeutic target for AD.

In AD mouse models, either pharmacological inhibition of sEH or genetic deletion of *Ephx2* significantly reduces Aβ deposition. Elevated EET levels not only diminish Aβ aggregation in the hippocampus and protect neurons from Aβ toxicity, but also promote Aβ clearance by enhancing lysosome biogenesis. These effects are closely associated with improved cognitive performance and reduced astrocyte activation, underscoring the importance of EETs in maintaining neuronal health and countering neurodegeneration [94].

Thus, EETs play a key role in AD pathology by alleviating Aβ‐induced mitochondrial dysfunction, reducing Aβ accumulation, and enhancing neuroprotection and lysosomal function. The relevance of EET signaling extends to other cognitive disorders; for instance, in models of diabetes‐associated cognitive impairment, sEH inhibition prolongs EET half‐life, reduces oxidative stress, inflammation, and apoptosis, and markedly improves cognitive function following diabetes‐induced hippocampal injury [95]. The broad anti‐inflammatory and anti‐apoptotic properties of EETs help mitigate neuronal injury and support the repair of damaged neurons, thereby preserving cognitive function across multiple neuropathological conditions—particularly those involving stress and inflammatory processes linked to cognition [96].

## 11 EETs in Psychiatric Disorders

Emerging evidence indicates that epoxyeicosatrienoic acids (EETs) play significant roles in psychiatric disorders—particularly depression and psychotic conditions—by exerting anti‐inflammatory, neuroprotective, and neural signaling‐modulating effects [97]. In the medial prefrontal cortex (mPFC), EET signaling has been implicated in depressive pathology. Mouse models of depression exhibit increased soluble epoxide hydrolase (sEH) activity, which negatively correlates with depressive‐like behaviors. Genetic deletion of *Ephx2*, the gene encoding sEH, enhances stress resilience and reduces such behaviors, while pharmacological sEH inhibition produces rapid antidepressant‐like effects, underscoring a direct link between EET pathway regulation and depressive states [98].

EET signaling counteracts depressive‐like behavior partly by regulating ATP release from astrocytes. Under depressive conditions, astrocyte function is substantially impaired by stress; EETs can modulate these alterations via ATP‐mediated mechanisms to alleviate stress‐induced symptoms. Both *Ephx2* knockout and sEH inhibition help restore normal astrocyte function. Additionally, EETs exhibit therapeutic potential through their anti‐inflammatory properties: sEH inhibition reduces neuroinflammatory responses in depression models, thereby ameliorating behavioral deficits. This effect is characterized by suppressed pro‐inflammatory factor release and enhanced neural stress resilience [98].

Beyond depression, EETs and their metabolites demonstrate broad anti‐inflammatory activity relevant to multiple psychiatric disorders. sEH inhibitors have been shown to potentiate EET‐mediated anti‐inflammatory effects and elicit both antidepressant‐ and antipsychotic‐like activities in preclinical models, suggesting their potential as a novel class of psychotropic therapeutics [99]. EETs also provide neuroprotection by modulating ion channels and intracellular signaling pathways. Through reduction of oxidative stress and apoptosis, EETs support overall brain function and strengthen stress adaptability. Their influence on neurotransmission may extend to multiple systems, including GABAergic signaling, thereby mitigating symptom severity across psychiatric illnesses [96].

The protective role of EETs in psychiatric disorders (primarily depression) is mainly mediated through the EET‐sEH signaling pathway in astrocytes, with core mechanisms including the regulation of astrocytic ATP release, suppression of neuroinflammation, and modulation of liver–brain axis signaling [100]. However, the role of the EET‐sEH pathway in other psychiatric disorders such as schizophrenia and anxiety disorders currently remains largely at the level of review‐based summaries, lacking direct evidence from original research. More independent original studies are needed in the future for validation.

## 12 EETs in Neuropathic Pain

In recent years, the role of EETs and their regulatory enzyme sEH in neuropathic pain has garnered significant attention, revealing an intriguing complexity: while most EET regioisomers (14,15‐EET, 11,12‐EET, 8,9‐EET) exhibit clear analgesic effects, 5,6‐EET mediates hyperalgesia via activation of the TRPA1 channel [101, 102]. The dual role of EETs in pain modulation exhibits noteworthy regioisomer‐specific characteristics, a key finding in this field in recent years. Among these EETs, 14,15‐EET has been the most extensively studied. In a rat model of central post‐stroke pain (CPSP) following thalamic hemorrhage, early administration of 14,15‐EET significantly inhibited glial activation, reduced the expression of pro‐inflammatory cytokines and apoptosis‐related proteins, and effectively alleviated mechanical allodynia. This study explicitly stated that the antinociceptive effect of 14,15‐EET is mediated, at least in part, by suppressing neuroinflammation and apoptosis [103]. Another study demonstrated that exogenous EETs effectively reduce mechanical allodynia in CPSP rats, with a faster onset and better analgesic effect than the current first‐line drug gabapentin [104].

The analgesic mechanisms of EETs involve multifaceted signaling regulation. At the spinal level, EETs or sEH inhibition produce anti‐hyperalgesic effects through two mechanisms: inhibiting the induction of cyclooxygenase‐2 (COX‐2) and rapidly promoting neurosteroidogenesis via upregulation of the steroidogenic acute regulatory protein (StARD1) [105]. Additionally, EETs can activate the endogenous opioid system and GABAergic system, thereby providing superior pain relief compared to morphine or gabapentin [106].

Given the analgesic effects of EETs and their rapid degradation by sEH, inhibitors targeting sEH have emerged as a highly promising therapeutic strategy for neuropathic pain. Extensive preclinical evidence has confirmed the efficacy of sEH inhibition in both inflammatory and neuropathic pain models with fewer side effects compared to many conventional analgesics [107, 108] For instance, research based on a thalamic hemorrhage CPSP rat model found that the sEH inhibitor TPPU significantly increased the mechanical paw withdrawal threshold, reduced the expression of endoplasmic reticulum stress markers and MAPK inflammatory signals (p‐p38 and p‐JNK), and inhibited the activation of perilesional microglia and astrocytes. This analgesic effect was completely abolished by the EET antagonist 14,15‐EEZE. The study further proposed that a vicious cycle between endoplasmic reticulum stress and neuroinflammation constitutes a key mechanism for central sensitization in CPSP, and that the EET/sEHi axis exerts analgesia by interrupting this crosstalk [109]. The novel sEH inhibitor AMHDU exhibited significant analgesic activity in a diabetic tactile allodynia model (10 mg/kg i.p., *p* < 0.05), with analgesic efficacy comparable to gabapentin but devoid of addiction risk due to distinct mechanisms. It also demonstrated low acute oral toxicity (> 2000 mg/kg) and a high therapeutic index [107]. Chemotherapy‐induced peripheral neuropathy (CIPN) is a major dose‐limiting factor in cancer treatment. In 2025, a novel sEH inhibitor, FP9 (IC_50_ = 0.2 nM), demonstrated significant and sustained analgesic effects in a paclitaxel‐induced CIPN mouse model, outperforming gabapentin, with an oral bioavailability of 78% [110]. These findings indicate that stabilization of epoxy fatty acids such as EETs represents a promising strategy for chronic pain management.

In recent years, researchers have begun exploring dual‐target inhibitors that combine sEH with other targets. In 2024, the sEH/HDAC6 dual‐target inhibitor M9 was reported to exhibit potent analgesic effects in vivo with better analgesic tolerance than gabapentin, becoming the first sEH/HDAC6 dual‐target inhibitor with in vivo anti‐neuropathic pain and anti‐inflammatory activity [111].

Research on EETs in neuropathic pain highlights two noteworthy trajectories: first, the functional divergence of regioisomers—5,6‐EET mediates pronociceptive effects via TRPA1, whereas 8,9‐, 11,12‐, and 14,15‐EET exert analgesic effects through multiple mechanisms. This “dual role” reveals the exquisite complexity of EET signaling. Second, the clinical translation of sEH as a therapeutic target—sEH inhibitors demonstrate robust analgesic effects and disease‐modifying potential in various neuropathic pain models, including CPSP, CIPN, and diabetic neuropathy.

Current challenges include: improving the clinical translational efficiency of sEH inhibitors, as the limitations of single‐target strategies suggest that multi‐targeting or multimodal approaches may be more promising; further elucidation of the precise functional localization and signaling pathways of different EET regioisomers within pain networks; and optimizing drug design to amplify EET‐mediated analgesia while avoiding potential side effects stemming from the 5,6‐EET‐TRPA1 axis. With the emergence of dual‐target inhibitors such as sEH/HDAC6 inhibitors and the continued clarification of EET receptor signaling pathways, this field holds promise for providing novel targeted therapeutic options for neuropathic pain.

## 13 EETs in Non‐Neurological Diseases

EETs play important regulatory and protective roles in a range of non‐neurological diseases, particularly in the cardiovascular system, cancer, metabolic disorders, and inflammatory conditions. In the cardiovascular system, EETs not only induce vasodilation to improve blood flow but also exert cardioprotective effects through anti‐inflammatory and anti‐fibrotic actions in settings such as atherosclerosis, hypertension, and heart failure. Inhibition of soluble epoxide hydrolase (sEH) prolongs the activity of EETs and enhances their anti‐inflammatory efficacy during cardiac remodeling [8]. Additionally, EETs help prevent thrombosis by inhibiting platelet aggregation and promoting fibrinolysis, thereby reducing the risk of thrombotic events including myocardial infarction and stroke [112]. Moreover, Nan et al. [113] revealed that 14,15‐EET attenuates sepsis‐induced cardiomyopathy by activating PPARα and inhibiting ferroptosis in cardiomyocytes.

In oncology, EETs contribute to primary tumor growth and multi‐organ metastasis by promoting angiogenesis and inhibiting apoptosis. This pro‐metastatic effect is not solely dependent on primary tumor proliferation but is also linked to the angiogenic actions of EETs at secondary sites, facilitating tumor dissemination. Consequently, inhibition of EET signaling can effectively suppress tumor growth and metastasis [114]. At the cellular level, EETs protect cancer cells from oxidative stress by upregulating antioxidant enzymes such as superoxide dismutase and catalase, thereby reducing mitochondrial damage and apoptosis. For instance, in arsenic trioxide (ATO)‐treated cancer cells, 11,12‐EET significantly attenuated ROS‐induced damage and cell death [115]. EETs also enhance endothelial cell migration and angiogenesis, further supporting tumor progression. These effects are often mediated through crosstalk with other signaling pathways, such as COX‐2, to augment tumor vascularization and blood supply [116]. Moreover, EETs are implicated in the development of drug resistance in breast cancer. Elevated CYP3A4‐derived 11,12‐EET levels in tamoxifen‐treated breast cancer cells have been observed, and disruption of this pathway can resensitize cells to treatment, highlighting EETs as a potential target in overcoming drug resistance [117].

In metabolic diseases, EETs help regulate lipid metabolism and improve insulin sensitivity. Studies demonstrate that EETs ameliorate metabolic parameters in conditions such as metabolic syndrome and diabetes, and by attenuating inflammatory responses, they also reduce associated cardiovascular risks [8]. In peripheral tissues such as the liver, EETs exert antioxidant and anti‐apoptotic effects that help maintain cellular homeostasis and protect against oxidative damage [118].

Taken together, these studies suggest broad roles for EETs in a wide spectrum of pathophysiological processes outside the nervous system, including inhibition of platelet aggregation, anti‐inflammatory and antioxidant protection, promotion of angiogenesis, tumor progression, suppression of apoptosis, and regulation of lipid metabolism. These multifaceted roles position EETs as potentially valuable targets, particularly in the context of drug‐resistant cancers and cardiometabolic diseases.

## 14 Conclusion and Prospections

**FIGURE 2:**
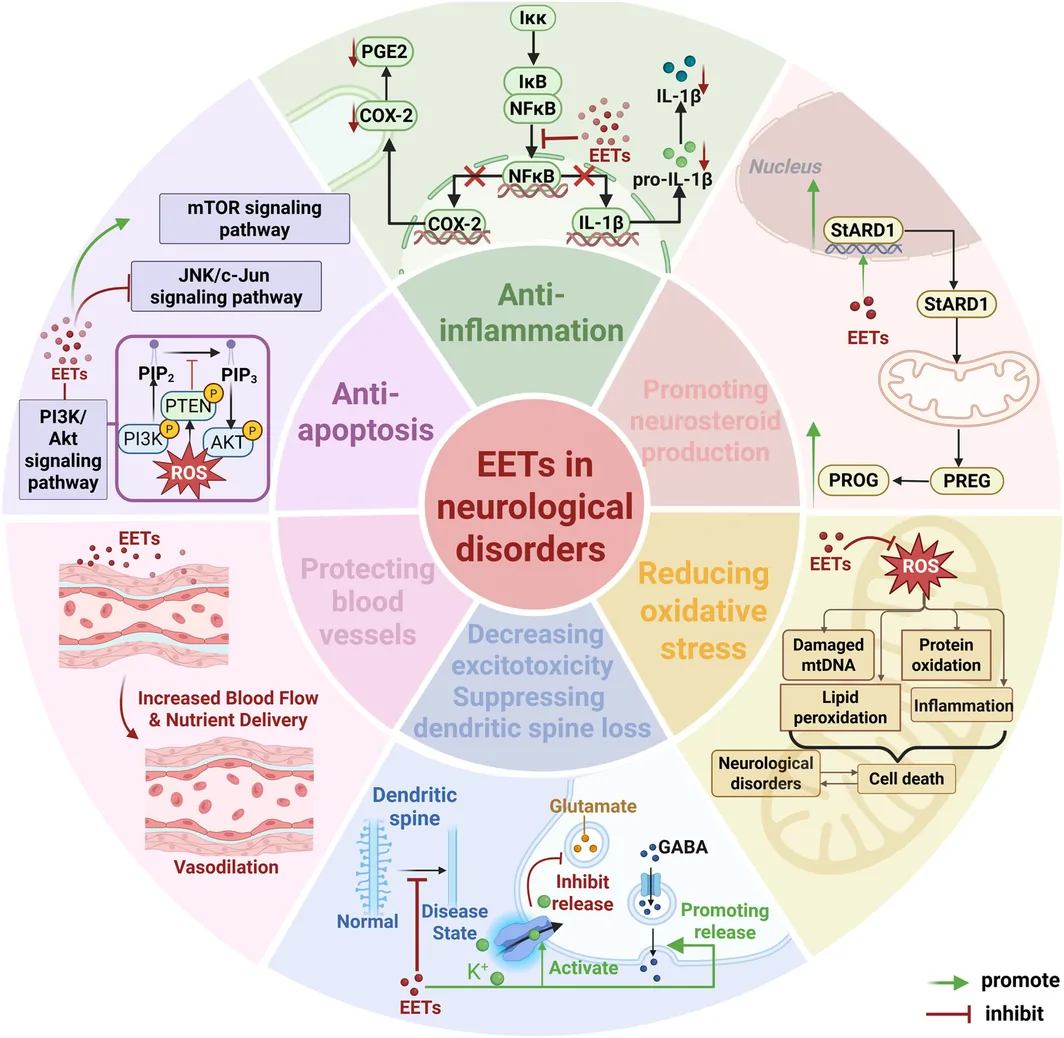
Pleiotropic mechanisms of EETs in neurological disorders. EETs ameliorate a range of neurological disorders—including cerebrovascular disease, epilepsy, Parkinson's disease, Alzheimer's disease, psychiatric disorders, and neuropathic pain—through diverse mechanisms. These include anti‐inflammatory and anti‐apoptotic effects, increased cerebral blood flow, reduced excitotoxicity, diminished dendritic spine loss, decreased oxidative stress, and the promotion of neurosteroid secretion.

EETs ameliorate a spectrum of neurological disorders—including cerebrovascular disease, epilepsy, Parkinson's disease, Alzheimer's disease, psychiatric conditions, and neuropathic pain—through multiple mechanisms. These include anti‐inflammatory and anti‐apoptotic effects, increased cerebral blood flow, reduced excitotoxicity, diminished dendritic spine loss, decreased oxidative stress, and enhanced neurosteroid secretion (Figure 2). In animal models, inhibition of soluble epoxide hydrolase (sEH), which elevates endogenous EET bioavailability, has been shown to slow the progression of various central nervous system (CNS) diseases and promote neuroprotection, thereby attenuating brain injury severity. Given these multimodal benefits, sEH inhibitors represent an attractive therapeutic strategy for a wide range of brain disorders.

However, when developing and evaluating sEH‐inhibitory therapies, it must confront several uncomfortable realities: the failure of lead compounds to progress beyond Phase II despite compelling preclinical data, the persistent BBB penetration gap that limits CNS applications, the fundamental gaps in understanding EET signaling mechanisms, and the narrowness of a single‐target inhibition strategy.

While theoretically attractive as an upstream strategy to enhance EET production, direct targeting of CYP epoxygenases poses formidable challenges. As fleming notes in a comprehensive review, “targeting CYP epoxygenases in vivo is difficult as these enzymes are involved in the metabolism of many currently used clinical agents” [119]. The human brain expresses CYP2J2 as the major epoxygenase, while multiple Cyp2j isoforms in mice confound translation [119]. Activators of CYP epoxygenases have not yet been successfully developed into drug candidates, and given their extensive role in xenobiotic metabolism, substantial off‐target risks are foreseeable. This avenue remains more valuable for mechanistic exploration than for near‐term drug development.

Moving forward, the community should prioritize: (1) rigorous validation of dual‐target sEH inhibitors with superior efficacy and safety profiles; (2) intensified efforts to identify and validate the cognate G protein‐coupled receptors (GPCRs) for EETs as a gateway to precise receptor pharmacology; (3) improved preclinical models that better predict clinical outcomes, with rat models showing superior translational relevance for sEH‐targeting therapeutics over mouse models; and (4) exploring peripherally restricted and non‐BBB‐dependent strategies (e.g., indirect CNS effects via peripheral anti‐inflammation)—and the ongoing STEP Study for EC5026 in PD will provide critical translational data to guide future directions.

## Author Contributions

H.Z., X.W., and J.D. contributed to the conception of the article. R.T. performed literature collection and drafted the manuscript. M.L. prepared the illustrations and table. B.H. and G.F. revised the manuscript. H.Z., X.W., and J.D. reviewed and edited the manuscript before submission. All authors approved the final version.

## Funding

This study was supported by grants from the Natural Science Foundation of Xiamen, China (No. 3502Z20227282); National Natural Science Foundation of China (Nos. 82171444, 82271499, 82271219, 91949129).

## Ethics Statement

The authors have nothing to report.

## Consent

The authors have nothing to report.

## Conflicts of Interest

The authors declare no conflicts of interest.

## Data Availability

The data that support the findings of this study are available from the corresponding author upon reasonable request.
